# STAT1: a novel candidate biomarker and potential therapeutic target of the recurrent aphthous stomatitis

**DOI:** 10.1186/s12903-021-01776-w

**Published:** 2021-10-14

**Authors:** Mingchen Cao, Lei Li, Long Xu, Mengxiang Fang, Xiaomin Xing, Changkai Zhou, Wei Ren, Longyuan Wang, Fanbo Jing

**Affiliations:** 1 Department of Pharmacy, The Affiliated Hospital of Qingdao University, Qingdao, China; 2 Department of Pharmacy, Huang Dao District Second Hospital of Traditional Chinese Medicine, Qingdao, China

**Keywords:** The recurrent aphthous stomatitis, WGCNA, Hub genes, STAT1, Reverse molecular docking

## Abstract

**Background:**

The recurrent aphthous stomatitis (RAS) frequently affects patient quality of life as a result of long lasting and recurrent episodes of burning pain. However, there were temporarily few available effective medical therapies currently. Drug target identification was the first step in drug discovery, was usually finding the best interaction mode between the potential target candidates and probe small molecules. Therefore, elucidating the molecular mechanism of RAS pathogenesis and exploring the potential molecular targets of medical therapies for RAS was of vital importance.

**Methods:**

Bioinformatics data mining techniques were applied to explore potential novel targets, weighted gene co-expression network analysis (WGCNA) was used to construct a co-expression module of the gene chip data from GSE37265, and the hub genes were identified by the Molecular Complex Detection (MCODE) plugin.

**Results:**

A total of 16 co-expression modules were identified, and 30 hub genes in the turquoise module were identified. In addition, functional analysis of Hub genes in modules of interest was performed, which indicated that such hub genes were mainly involved in pathways related to immune response, virus infection, epithelial cell, signal transduction. Two clusters (highly interconnected regions) were determined in the network, with score = 17.647 and 10, respectively, cluster 1 and cluster 2 are linked by STAT1 and ICAM1, it is speculated that STAT1 may be a primary gene of RAS. Finally, genistein, daidzein, kaempferol, resveratrol, rosmarinic acid, triptolide, quercetin and (-)-epigallocatechin-3-gallate were selected from the TCMSP database, and both of them is the STAT-1 inhibitor. The results of reverse molecular docking suggest that in addition to triptolide, (-)-Epigallocatechin-3-gallate and resveratrol, the other 5 compounds (flavonoids) with similar structures may bind to the same position of STAT1 protein with different docking score.

**Conclusions:**

Our study identified STAT1 as the potential biomarkers that might contribute to the diagnosis and potential therapeutic target of RAS, and we can also screen RAS therapeutic drugs from STAT-1 inhibitors.

## Background

Recurrent aphthous stomatitis (RAS) is recognized as the most common oral mucosal disease [1]. RAS is a painful (include prodromal burning sensation), well-circumscribed, and round-shaped ulceration that is covered by a yellow-grayish pseudomembrane and surrounded by an erythematous halo, or become confluent to produce larger, plaqueform, irregular lesions throughout the oral cavity [2, 3]. The classic presentation of RAS is recurrent, self-limiting ulcers that mainly affect nonkeratinized oral mucosa (typically located on the buccal, labial mucosa, tongue and floor of the mouth). Involvement of the heavily keratinized mucosa of the palate and gingiva is less common [4]. Since oral disorders frequently have detrimental effects on speech, nutrition, physical appearance, self-esteem and social interaction, especially RAS frequently affects patient quality of life as a result of long lasting and recurrent episodes of burning pain [5].

Although the molecular mechanism of RAS pathogenesis is not yet clear, it may involve biological processes such as immune response, chronic inflammation, oxidative stress, extracellular matrix, et al. [5–7]. Therefore, elucidating the molecular mechanism of RAS pathogenesis and exploring the potential molecular targets of medical therapies for RAS is of vital importance. With the widespread application of gene chips and high-throughput sequencing technologies, databases related to genomes have accumulated a large amount of data [8]. Computerization methodologies have been applied into the discovery of signature genes as potential biomarkers of diseases [9]. How to use bioinformatics technology to deeply explore the potential value of these data has become one of the important directions for studying the molecular mechanisms of diseases. The bioinformatics analysis methods can help us study the molecular mechanism of diseases and discover potential therapeutic targets from a systematic perspective [8, 9]. Among all the bioinformatics analysis methods, the weighted gene co-expression network analysis (WGCNA) is a useful advanced and comprehensive algorithm approach for the analysis of the gene expression patterns of multiple samples [10]. The unique advantage of WGCNA is the ability to analyzes gene expression profiling to cluster genes and form co-expression modules by similarly behaving genes with a common biological relationship or function, that reveal the gene networks and signaling pathways and identify intramodular hub genes [11]. It has been successfully used to study various biological processes, proving to be quite helpful for the identification of candidate biomarkers and potential therapeutic targets [10, 11].

Management of RAS depends upon the frequency and severity of the lesions [5]. Most RAS cases can be adequately managed with topical therapy, the current treatment methods include pain relief, anti-inflammatory, and promotion of ulcer healing, while mainly include antibiotic therapy, hormonal therapy, medicine mouthwash, and laser therapy [5–7]. However, there are temporarily few available effective medical therapies to treat RAS currently. Traditional Chinese medicine has accumulated many natural medicines for the treatment of diseases, molecular biology and drug molecular target identification techniques have been more and more widely used in current Chinese herbal medicine research [12]. Drug target identification, which includes many distinct algorithms for finding genes and proteins, is the first step in drug discovery, the problem of target identification is usually finding the best interaction mode between the potential target candidates and probe small molecules [13]. Many computer simulation analysis technologies have been developed for the confirmation of lead compounds, such as structure-based target discovery methods (such as pharmacophores, similar binding sites, fingerprint-based interaction methods, and molecular docking), representative databases such as TCMSP, Pharmmapper and others, calculate and save a large number of target data of natural active chemical components [12–14].

In this study, we used a variety of bioinformatics analysis tools to conduct in-depth data mining on the gene chip data of 28 RAS patient samples, and finally determined that STAT1 may be a key target affecting the RAS process and a potential therapeutic target at the same time, based on this target, the natural chemical components of 8 herbs were screened, which may become potential drugs for local treatment of RAS, providing new directions for follow-up research.

## Methods

### Data Collection and Validation of the datasets

The gene expression dataset used for our analysis was screened from the Gene Expression Omnibus (GEO) database (http://www.ncbi.nlm.nih.gov/geo/), “recurrent aphthous stomatitis” was used as the search keyword. A dataset, with a GEO tracking number GSE37265 and a platform entry number GPL570 provided by Baccaglini L et al., was screened out and download. Sample collection and microarray dataset were performed by the Microarray lab 103, Molecular Genetics and Microbiology, University of Florida. In this dataset, transcription profiles were established from normal tissue from control individuals and ulcer and non-ulcer tissue from afflicted individuals. The transcriptional profiles were measured by Affymetrix Human Genome U133 Plus 2.0 Array.

### Differential expression genes (DEGs) analysis

The matrix file was annotated with an official gene symbol using the data table of the microarray platform, the “sva” R package was used to conduct batch normalization of the original expression data, and a normalized gene expression matrix file containing data was obtained for DEGs analysis. The “limma” R package was used to conduct DEGs analysis. The *P*-value of genes was calculated using t test method, and Benjamini and Hochberg's method was used to calculate the adjusted *P*-value.

### Construct the miRNA-gene interaction network

MicroRNA (miRNA) are identified to play a key role in regulating development in mammalian organisms. The analysis of miRNA and protein coding genes were studied based on the TarBase (http://www.microrna.gr/tarbase), the largest available manually curated target database, indexed targets derived from high throughput experiments, provides millions of high quality manually curated experimentally validated miRNA-gene interactions[15].

### Construct the signaling information network

The regulation of gene and protein expression in organisms is inseparable from the extensive participation of chemicals such as signaling molecule, the signaling information network was constructed based on the SIGNOR 2.0 (https://signor.uniroma2.it/), a public repository that stores manually-annotated causal relationships between proteins and other biologically relevant entities (chemicals, phenotypes, complexes, etc.) that participate in signal transduction relationship, represented graphically as a signed directed graph[16].

### Weighted gene Co-Expression network analysis and co-expression network construction

The weighted gene correlation network analysis was performed to construct a co-expression network via R (3.6.2) WGCNA package, a typical system biology algorithm. First, we performed cluster analysis of the samples to detect the outliers by the hclust function [10, 11].

### Gene Set Enrichment Analysis (GSEA) of gene modules

The constructed modules were consisted of a number of genes and functional enrichment analysis was then performed on the DEGs in those modules. To obtain the biological functions and signaling pathways involved in those modules, DEGs in modules were subjected to gene ontology (GO) analysis and (KEGG) pathway analysis using the GSEA software (GSEA version 4.0.3) [17]. After multiple test calibration, we used “adjusted *P* < 0.002” and “FDR < 0.05” as the threshold value to identify the enriched terms, and the top 10 most important terms were screened.

### PPI network

A PPI network was constructed to evaluate the interactions between genes, which helps us to explore novel molecular mechanism. Modules of interest were visualized using STRING 11.0 (https://string-db.org/), an online database to search the interaction among different proteins [18]. The common genes in the preserved modules that were obtained from WGCNA, and the DEGs with significant consistency, were selected to construct a PPI network, visualized using Cytoscape 3.7.2 software [19]. In the PPI network, a node represents a gene; the undirected link between two nodes is an edge, denoting the interaction between two genes; and the degree of a node corresponds to the number of interactions of a gene with other genes in the network, and only experimentally validated interactions with a combined score of more than 0.9 were selected as significant. Using node degree and interaction score as the key topological parameters, the maximally connected genes were informally referred to as hub genes.

### Identification and validation of hub genes

The intra-module connectivity of a gene is equal to the sum of the degree of correlation between this gene and other genes in that module. The top 30 genes with the highest intra-module connectivity were selected as hub genes. After screening out the interested modules, the weighted gene co-expression network was constructed using Cytoscape, and the hub genes were identified by the Molecular Complex Detection (MCODE) plugin. Gene regulatory network could help us accurately screen candidate genes that were potentially involved in the regulation of target genes, and could use the function of known genes to predict unknown gene function.

### Screening of active ingredients of natural medicines acting on hub genes

TCMSP is a pharmacology platform of Chinese herbal medicines that focus on the exploration of the active ingredients and targets, which had collected 499 herbs, with a total of 12,144 chemicals, as well as pharmacokinetic properties for natural compounds [20]. The drug-target were obtained from two sources: (1) experimental validated drug-target pairs were retrieved from HIT database (2) the SysDT model constructed was used to predict the potential targets of a compound [20]. In order to obtain the related ingredients based on the TCMSP database, we selected the search category as "targets name" and the keyword setting as "signal transducer and activator of transcription 1-alpha/beta " to search, DL ≥ 0.1 as the filter condition.

### Predict potential targets

PharmMapper[21] is designed to identify potential target candidates for the given probe small molecules using pharmacophore mapping approach. Upload Query File:.Mol2, parameter set: Generate Conformers: Yes; Maximum Generated Conformations: 300; Select Targets Set: Druggable Pharmacophore Models (v2017, 16,159); Number of Reserved Matched Targets (Max 1,000): 500. After submitting and waiting for the calculation to be completed, the results are saved in csv file format.

### Reverse molecular docking verification

Molecular docking was performed by AutoDock Vina [22]. All visualizations of biomolecules were conducted by PyMol Software [23].

### Statistical tests

By convention in biology, *P* ≤ 0.05 is considered the cutoff for statistical significance.

## Results

### Validation of the datasets

**Fig. 1:**
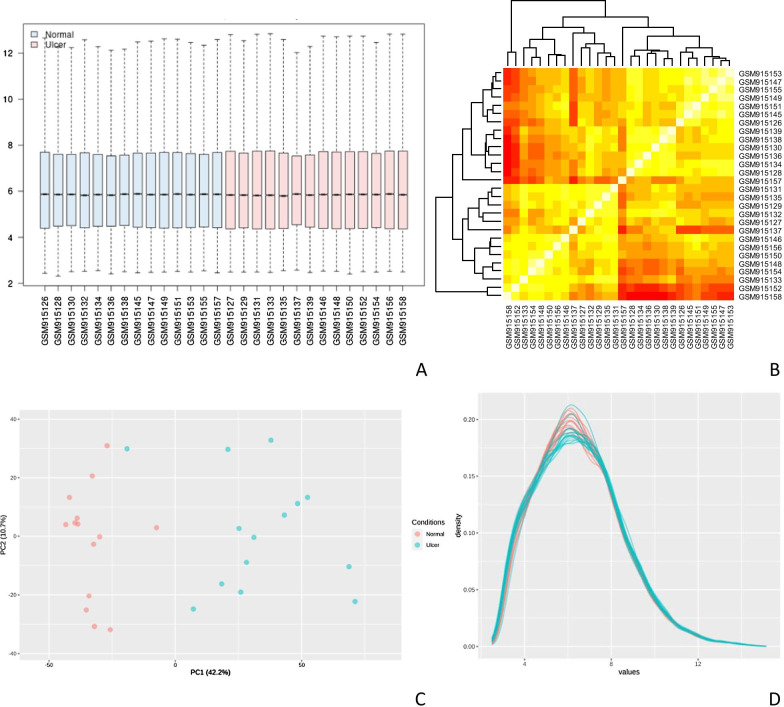
**A** Box plot showing distribution of raw read counts in the GSE37265 dataset, **B** Pearson’s correlation analysis of samples from the GSE37265 dataset. **C** PCA of samples from the GSE37265 dataset. **D** The diagnostic plot

We normalized the raw data of GSE37265 before analysis, the box plot showing distribution of raw read counts in the dataset (Fig. 1A). To further validate the intra-group data repeatability, we employed the Pearson’s correlation test and principal component analysis (PCA). The color reflects the intensity of the correlation, when 0 < correlation < 1, there exists a positive correlation. When − 1 < correlation < 0, there exists a negative correlation, the larger the absolute value of a number the stronger the correlation, which showed that there were strong correlations among the samples in the health group and RAS group in the GSE37265 dataset (Fig. 1A). Based on the PCA, the intra-group data repeatability for GSE37265 dataset was acceptable. In the PCA diagram, principal component 1 (PC1) and principal component 2 (PC2) are used as the X-axis and Y-axis, respectively, to draw the scatter diagram, where each point represents a sample, the farther the two samples are from each other, the greater the difference is between the two samples in gene expression patterns. The distances between per samples in the control group and the recurrent aphthous stomatitis group were acceptable in the dimension of principal component-1 (PC1) (Fig. 1B). The diagnostic plot summarizing the standard deviation versus mean measures of reads in the samples for each gene, which showed the dependence between counts and variance was acceptable. The plot of density against log2 of read counts displays the relative distribution of different counts in the health group and RAS group.

### Differentially expressed genes (DEGS) between RAS and healthy control

**Fig. 2:**
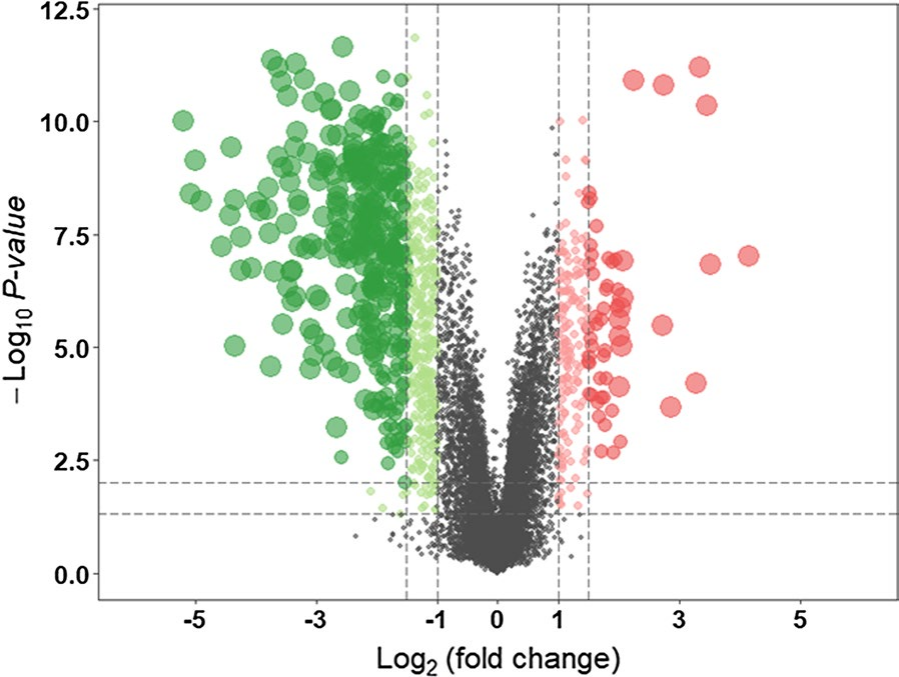
The volcano plot shows the up-regulated and down-regulated genes in RAS. The horizontal axis represents the fold change between health and RAS. The vertical axis represents the *P* value of t test for the differences between health and RAS

**Fig. 3:**
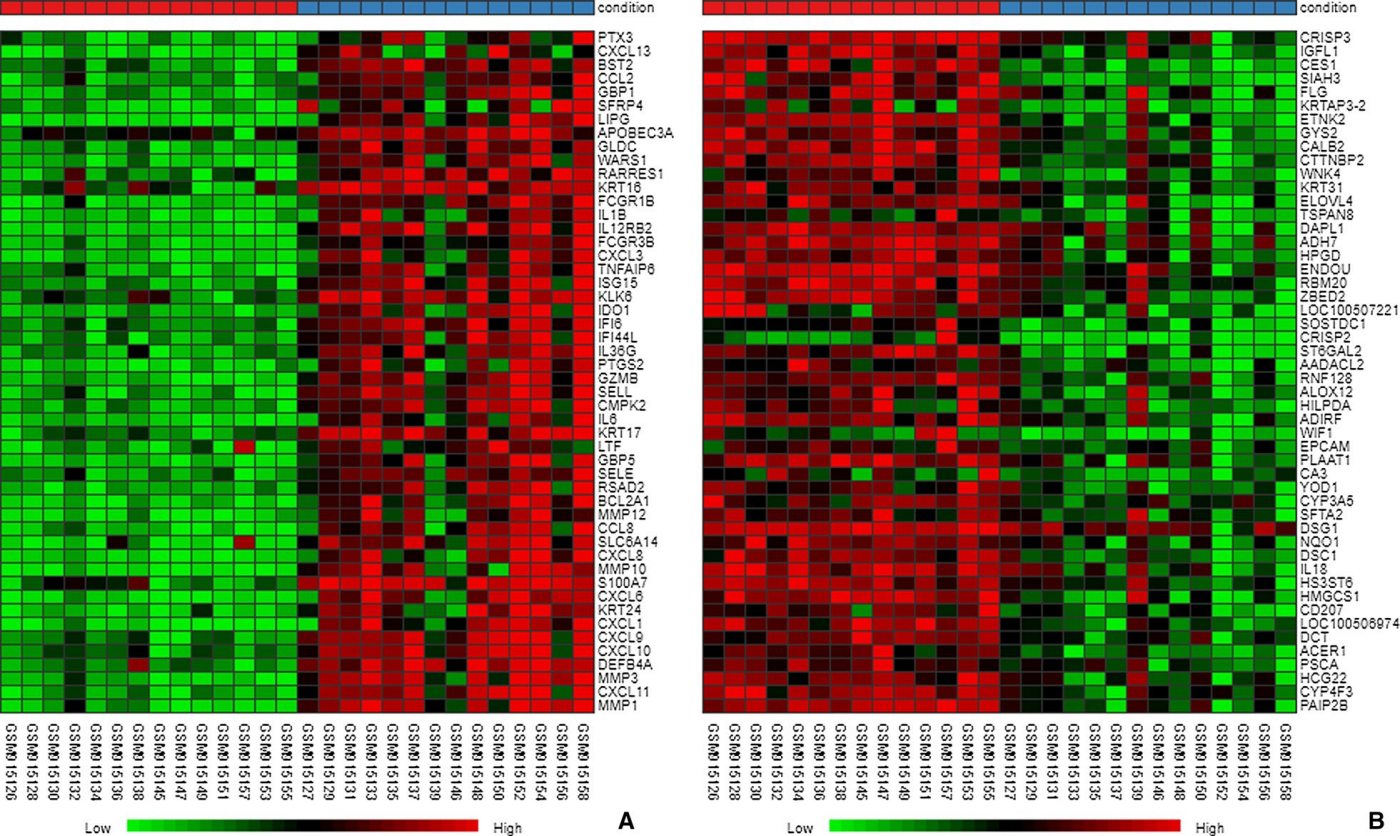
The heatmap shows the top 50 differential gene expression (*P* < 0.05) between health and RAS. **A** The heatmap of top 50 most significant down-regulated genes; **B** The heatmap of top 50 most significant upregulated genes

The recurrent aphthous stomatitis samples from cohort GSE37265 were analyzed using R software and its extension packages. The gene expression matrix was obtained after data preprocessing (included 12,548 genes). A total of 187 DEGs were identified with the threshold at |log2 (fold-change) | > 2 and *P* < 0.05, which consisted of 125 down-regulated genes and 18 up-regulated genes, and the volcano plot of all probesets is shown in Fig. 2. The 50 most significant down-regulated genes and up-regulated genes were visualized using a heatmap (Fig. 3). Red represents increased expression, whereas blue represents decreased expression. The most up-regulated genes included DAPL1, TSPAN8, ELOVL4, KRT31, WNK4, CTTNBP2, CALB2, GYS2, ETNK2, KRTAP3-2, whereas MMP1, CXCL11, MMP3, DEFB4A, CXCL10, CXCL9, CXCL1, KRT24, CXCL6, S100A7, MMP10, CXCL8, SLC6A14, CCL8, MMP12 were the most down-regulated genes in the RAS samples.

### Construct miRNA-gene interaction network

**Fig. 4:**
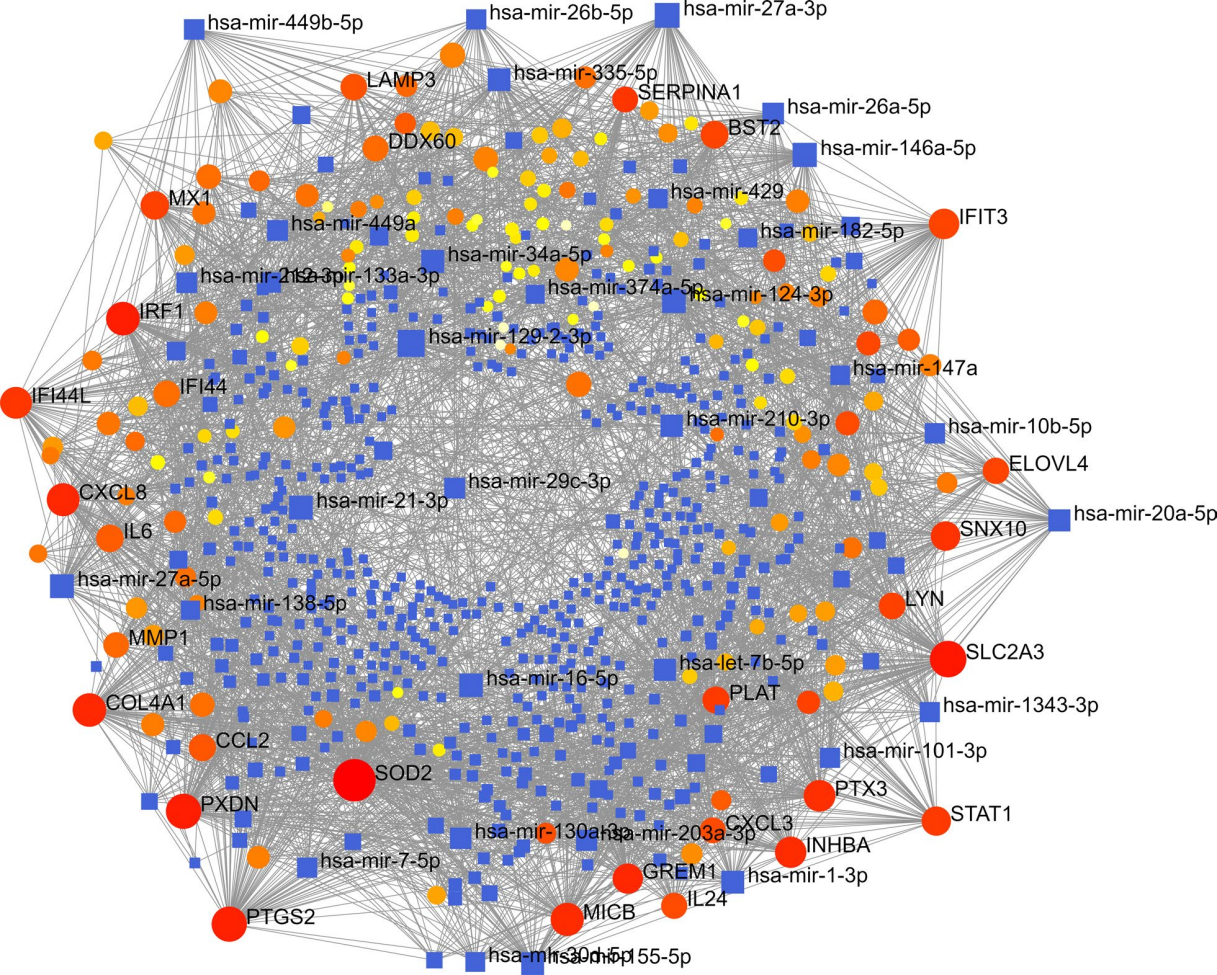
The miRNA-gene interaction network of RAS

As shown in the Fig. 4, we constructed a miRNA-gene interaction network of DEGs based on TarBase, Table 1 lists the top 20 high-level genes according to their interaction degrees, which reveals that SOD2, SLC2A3, PXDN, PTGS2, IRF1, COL4A1, MICB, CXCL8, etc. may play an important role in the miRNA regulatory network.

**Table 1:** The top 20 genes of miRNA-gene interaction network of RAS

Id	Label	Degree	Betweenness	Expression
6648	SOD2	156	53,322.71	− 2.45583
6515	SLC2A3	95	23,070.28	− 2.24469
7837	PXDN	85	19,402.04	− 2.22854
5743	PTGS2	85	17,813.26	− 3.55500
3659	IRF1	73	18,693.35	− 2.20671
1282	COL4A1	73	13,650.85	− 2.32325
4277	MICB	70	12,782.87	− 2.23157
3576	CXCL8	67	13,077.81	− 4.33586
10,964	IFI44L	62	8806.11	− 3.49043
5806	PTX3	61	11,559.62	− 3.03014
3624	INHBA	59	12,948.04	− 2.57143
3437	IFIT3	54	5974.13	− 2.93725
26,585	GREM1	53	13,463.77	− 2.47693
29,887	SNX10	49	10,198.16	− 2.62950
6772	STAT1	48	7937.42	− 2.06146
4599	MX1	44	6582.67	− 2.03886
684	BST2	41	5885.18	− 3.04879
3569	IL6	40	2793.91	− 3.67836
5327	PLAT	38	8060.72	− 2.85614
6347	CCL2	38	3233.80	− 3.07236

### Construct the signaling information network based on SIGNOR

**Fig. 5:**
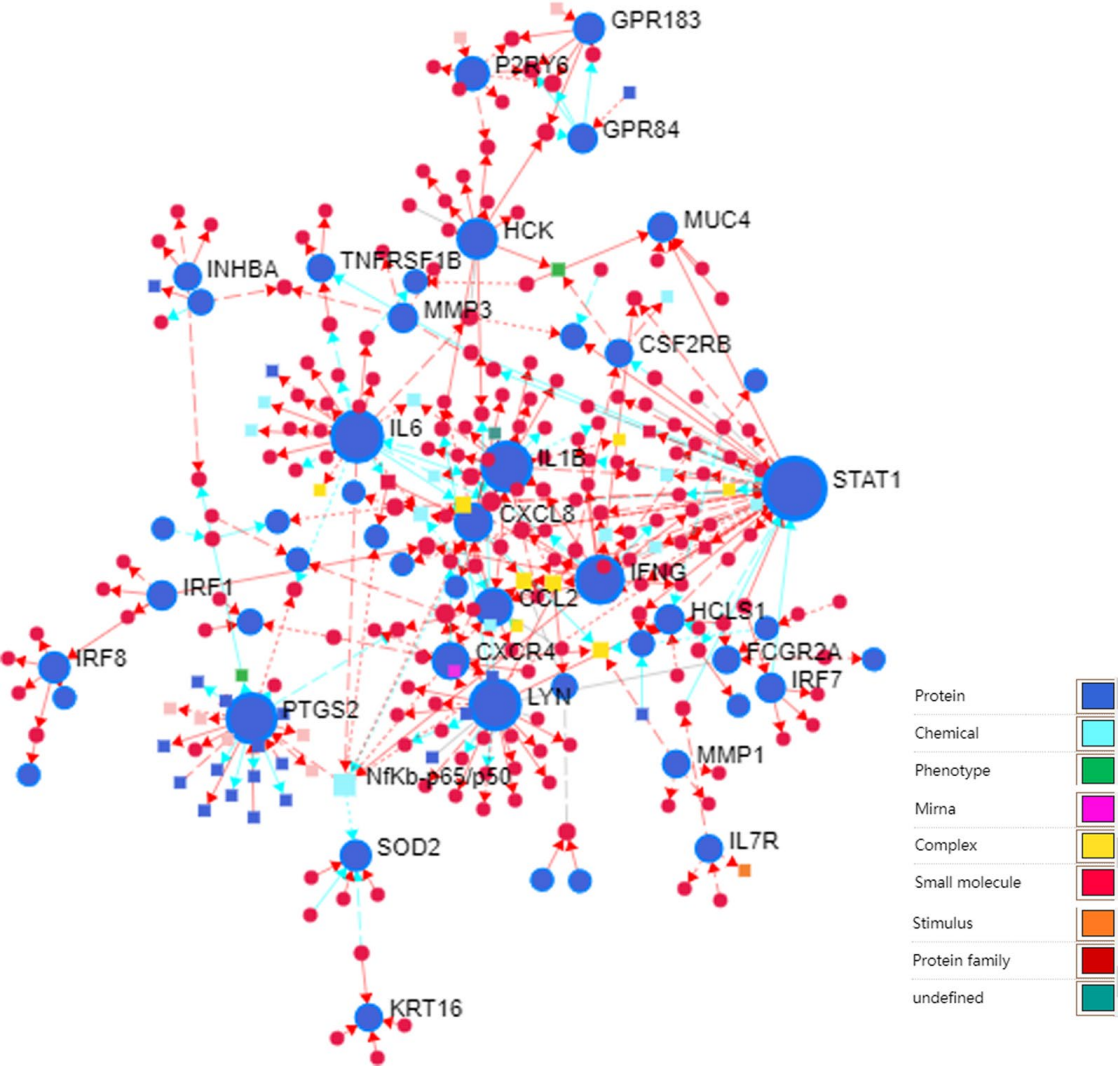
The signaling information network based on SIGNOR

As shown in the Fig. 5, we constructed the signaling information network of DEGs based on SIGNOR2.0, Table 2 lists the top 20 high-level genes according to their interaction degrees, which reveals that STAT1, IL6, LYN, PTGS2, IL1B, IFNG, HCK, CXCL8, CCL2, CXCR4, etc. participated extensively in the regulation of chemical signaling substances in this network.

**Table 2:** The top 20 genes of signaling information network of RAS

Id	Label	Degree	Betweenness	Expression
6772	STAT1	48	22,698.35	− 2.06146
3569	IL6	27	8361.34	− 3.67836
4067	LYN	26	8816.84	− 2.37319
5743	PTGS2	25	7101.68	− 3.55500
3553	IL1B	25	5464.20	− 3.32514
3458	IFNG	22	5069.17	− 2.72686
3055	HCK	13	6563.89	− 2.36321
3576	CXCL8	11	3444.47	− 4.33586
6347	CCL2	11	1569.33	− 3.07236
7852	CXCR4	10	3602.66	− 2.03314
5031	P2RY6	8	1444.38	− 2.05379
6648	SOD2	6	2492.00	− 2.45583
3394	IRF8	6	1676.00	− 2.35421
1880	GPR183	6	743.57	− 2.23414
3659	IRF1	5	2754.00	− 2.20671
4314	MMP3	5	1947.00	− 6.14321
3059	HCLS1	5	1258.00	− 2.28786
4585	MUC4	5	1204.32	− 2.07303
3665	IRF7	5	843.00	− 2.11757
53831	GPR84	5	602.76	− 2.15514

### WGCNA Co-Expression Network and Construction of coexpression modules

**Fig. 6:**
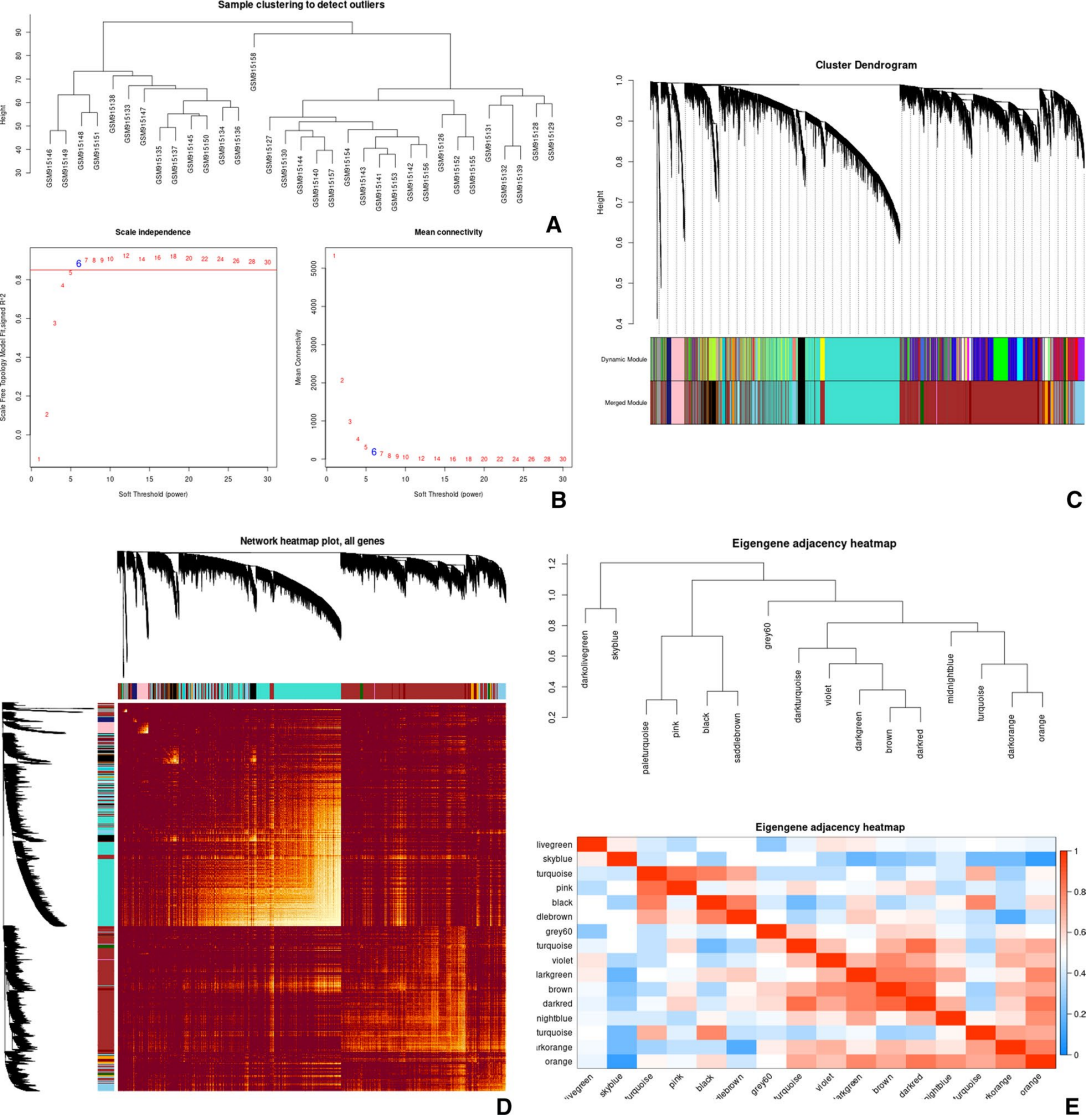
The result of WGCNA analysis. **A** Sample clustering to detect outliers; **B** Analysis of network topology for a set of soft‐thresholding powers. Scale independence and mean connectivity of various soft-thresholding values (β). The left picture displays the scale free fit index (y‐axis) as a function of the soft‐thresholding power (x‐axis). The right picture shows the mean connectivity (degree, y‐axis) as a function of the soft‐ thresholding power (x‐axis); **C** clustering dendrograms of genes, with dissimilarity based on topological overlap, together with assigned module colors. Cluster dendrogram of all filtered genes enriched based on the dissimilarity measure and the cluster module colors; **D** the heatmap plot describes the Topological Overlap Matrix (TOM) among DEGs in the analysis; **E** The eigengene dendrogram and heatmap identify groups of correlated eigengenes termed meta modules

We performed network topology analysis to determine candidate power values for relative, balanced scale independence, and mean connectivity in the WGCNA. As a result, the 6208 DEGs (adjust *P* values < 0.05) of the RAS samples were used to construct co-expression modules using the WGCNA algorithms. Subsequently, hierarchical clustering analysis was performed with the flashClust function and the results are presented in Fig. 6A. The soft-power threshold β was determined by the function “sft$powerEstimate”, as shown in Fig. 6B, a power value of 6 was the lowest power for which scale independence was below 0.8, and this was selected to produce a hierarchical clustering tree of the 6208 genes. Finally, 16 modules were identified based on average hierarchical clustering and dynamic tree clipping, each module had different color and genes. All the modules were significantly independent of each other, eigengene module values were calculated in each module and a clustering tree is presented in Fig. 6C. Among all the modules, the turquoise module had the highest number of hub genes. Then, gene modules were detected based on the TOM matrix, Interactions between the 16 modules were then analyzed (Fig. 6D). In addition, the eigengene dendrogram and heatmap were used to quantify module similarity by eigengene correlation (Fig. 6E).

### Define the DEGs in co-expression modules

Interestingly, we found that almost all of the differentially expressed genes, especially the key nodes in the above two networks analysis are involved in the Turquoise module, including SOD2, STAT1, PTGS2, IL-6, etc. Therefore, we use the R software to obtain the DEGs (log2 (fold-change) | > 1.5 and *P* < 0.05) of the Turquoise module, there are 254 DEGs among the 677 co-expressed genes in the Turquoise module.

### GSEA enrich analysis

**Fig. 7:**
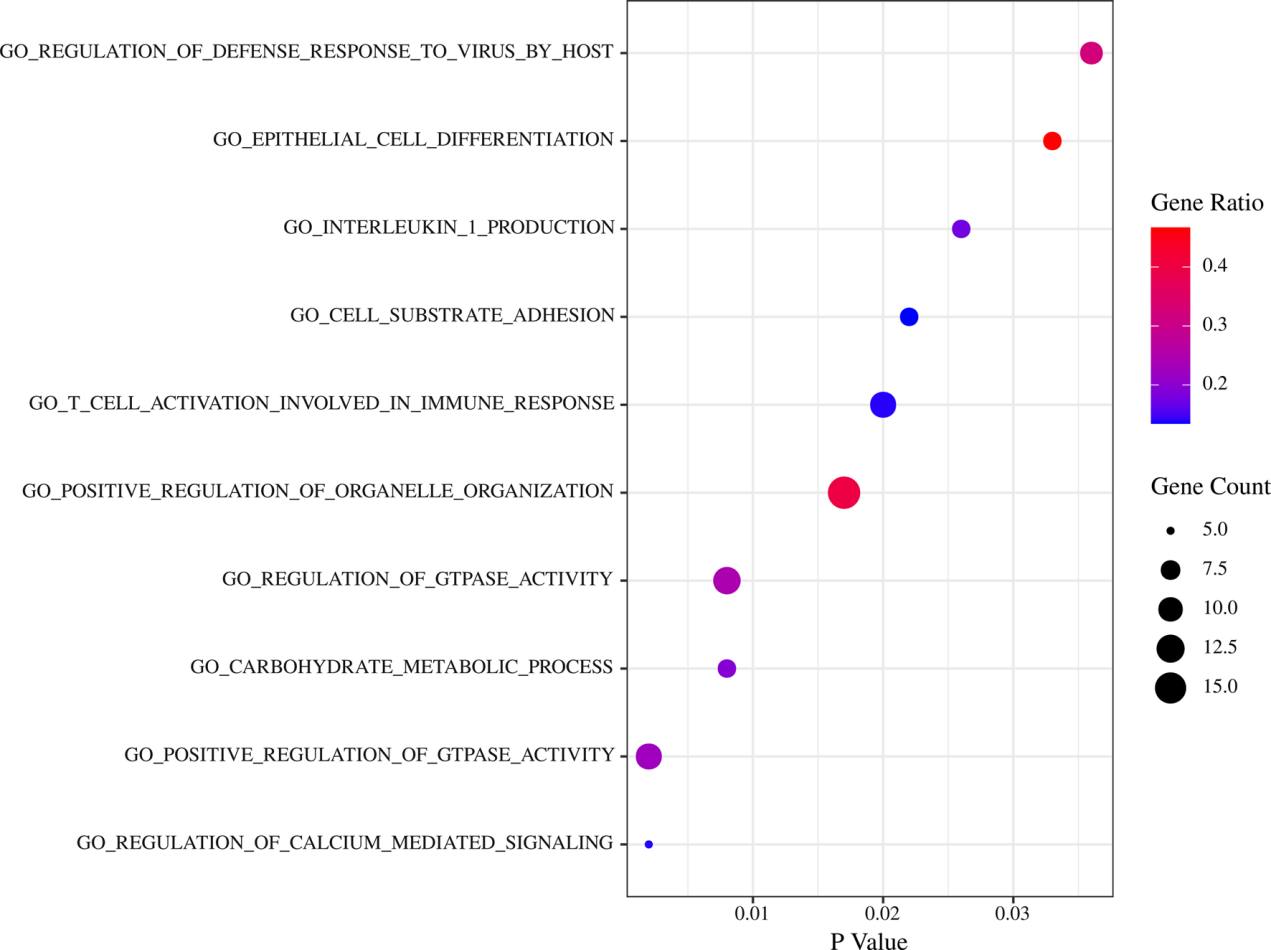
The result of GSEA enrichment analysis based on Go (biological process)

**Fig. 8:**
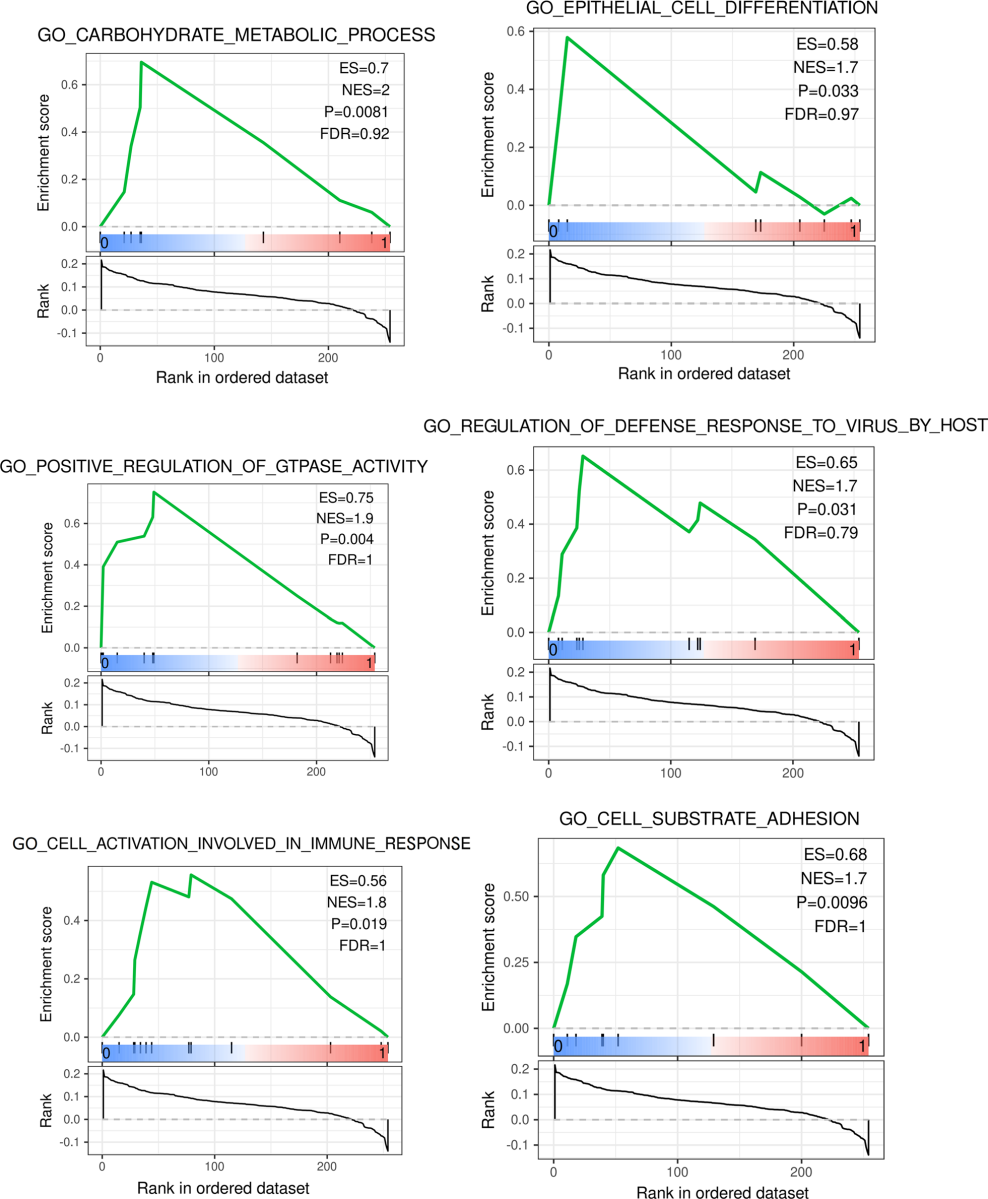
The Go (biological process) pathways highly related to RAS

The Fig. 7 showed the result of GSEA enrichment analysis based on Go (biological process). As shown in Fig. 8, The pathways for the DEGs in the Turquoise modules mainly focus on immune response, virus infection, epithelial cell, signal transduction, which the pathways that are highly related to RAS mainly include positive regulation of GTPase activity, T cell activation involved in immune response, epithelial cell differentiation, positive regulation of organelle organization, cell substrate adhesion, regulation of defense response to virus by host, regulation of calcium mediated signaling, interleukin 1 production, etc. A total of 56 core targets were enriched, including ICAM1, CCR7, IL1B, PLEK, CCL4, NCKAP1L, GPR65, ZC3H12A, P2RY6, CCL8, RGS1, CCL2, ARHGAP9, ADAP2, RGS18, ITGAL, LCP1, LILRB1, STAT1, MSN, CORO1A participates in more than 2 GO pathways, and the ICAM1 with the highest frequency which participates in 5 pathways.

**Fig. 9:**
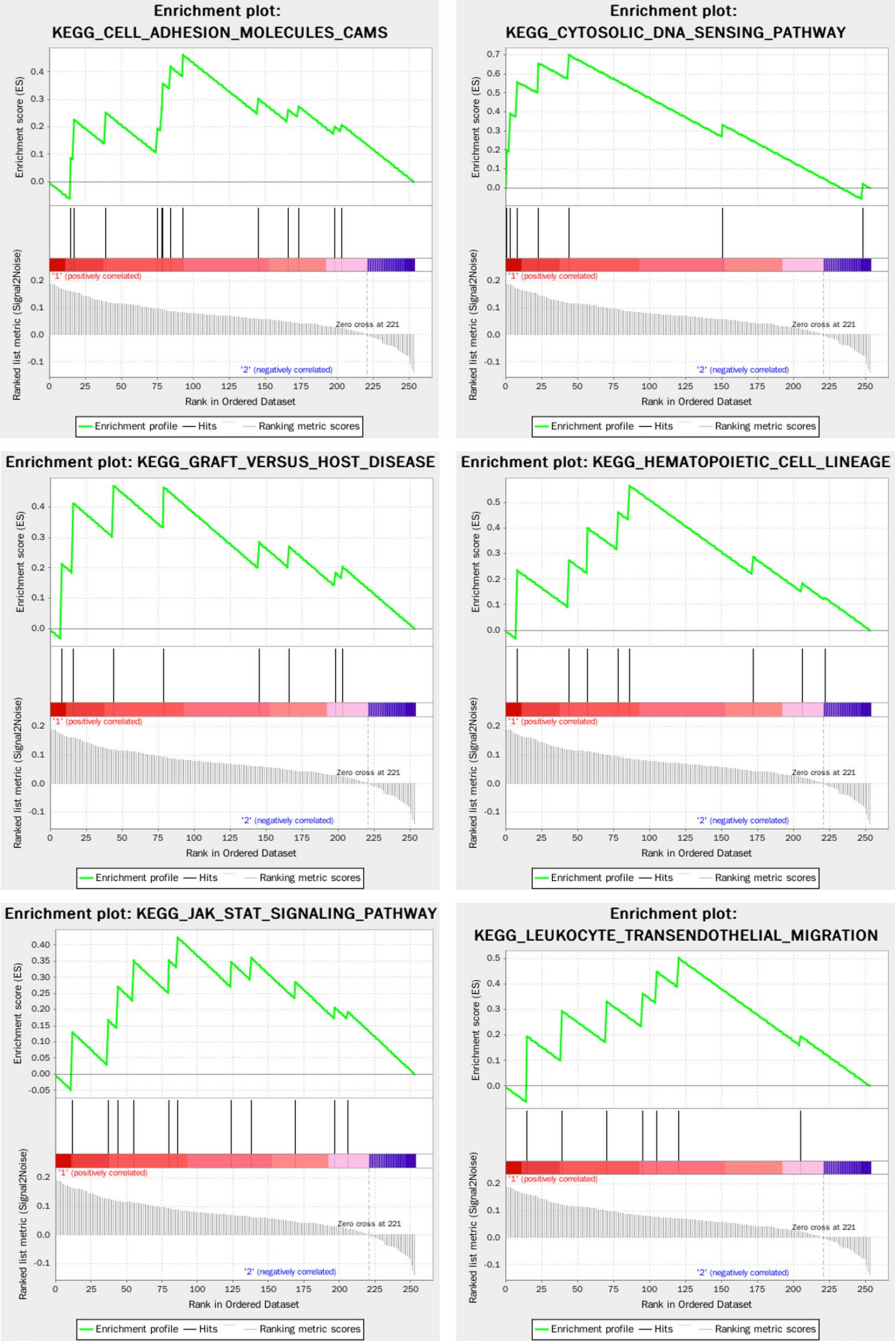

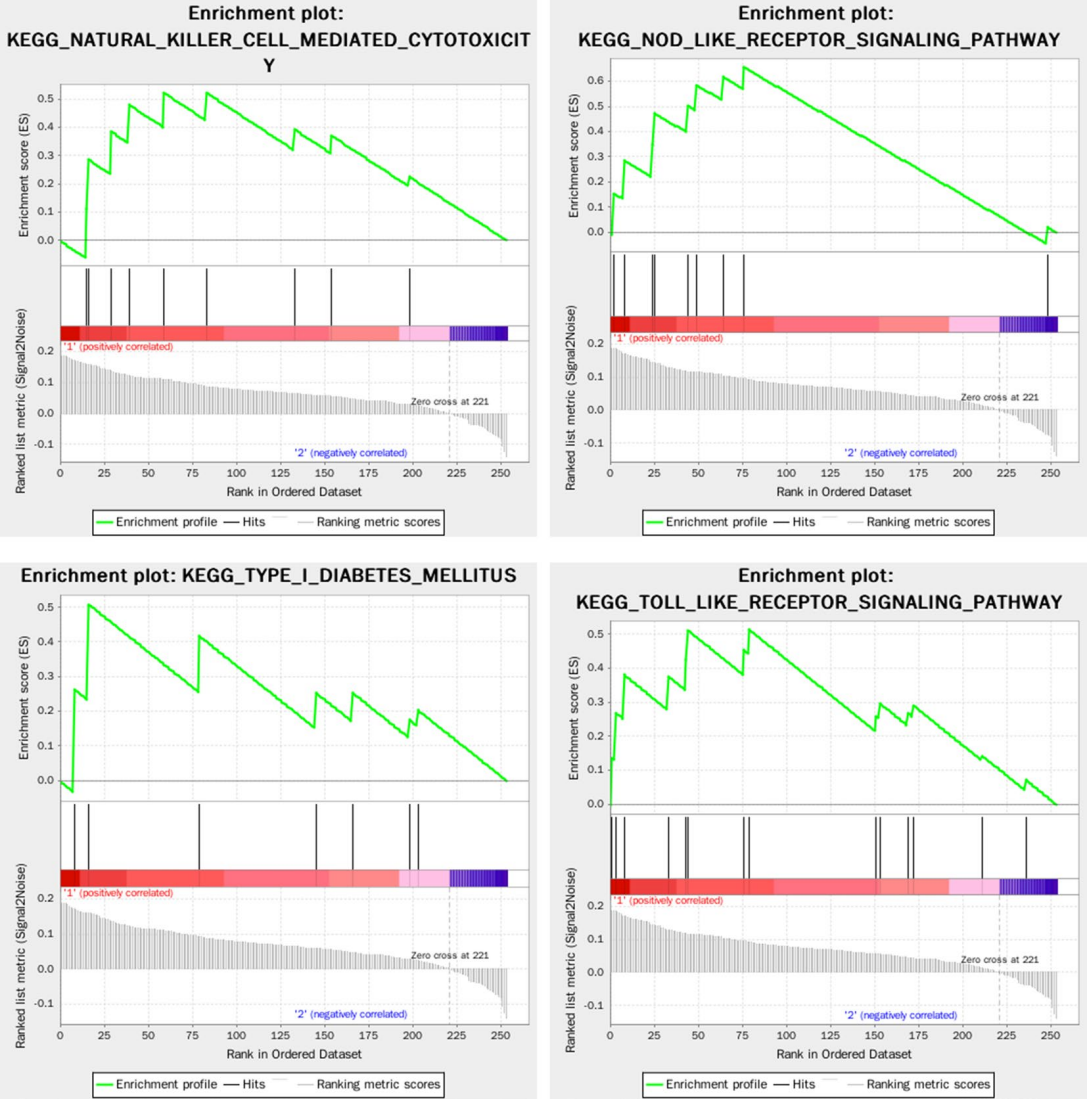
The result of GSEA enrichment analysis based on KEGG

The Fig. 9 showed the result of GSEA enrichment analysis based on based on KEGG. The pathways for the DEGs in the Turquoise modules that are highly related to recurrent RAS mainly include cell adhesion molecules cams, cytosolic DNA sensing pathway, natural killer cell mediated cytotoxicity, type I diabetes mellitus, nod like receptor signaling pathway, hematopoietic cell lineage, graft versus host disease, leukocyte transendothelial migration, JAK/STAT signaling pathway, TOLL like receptor signaling pathway, etc. A total of 55 key genes were enriched, including IL1B, IL6, CD86, HLA-B, ICAM1, ITGAL, HLA-DMA, HLA-F, HLA-DMB, GZMB, CD2, CCL4, CXCL10, IRF7, IL18, CXCL8, IL7R, CD14, CSF3R, STAT1 participates in more than 2 pathways, and the IL1B and IL6 target with the highest frequency participates in 6 pathways.

### Identification and Validation of Hub Genes

**Fig. 10:**
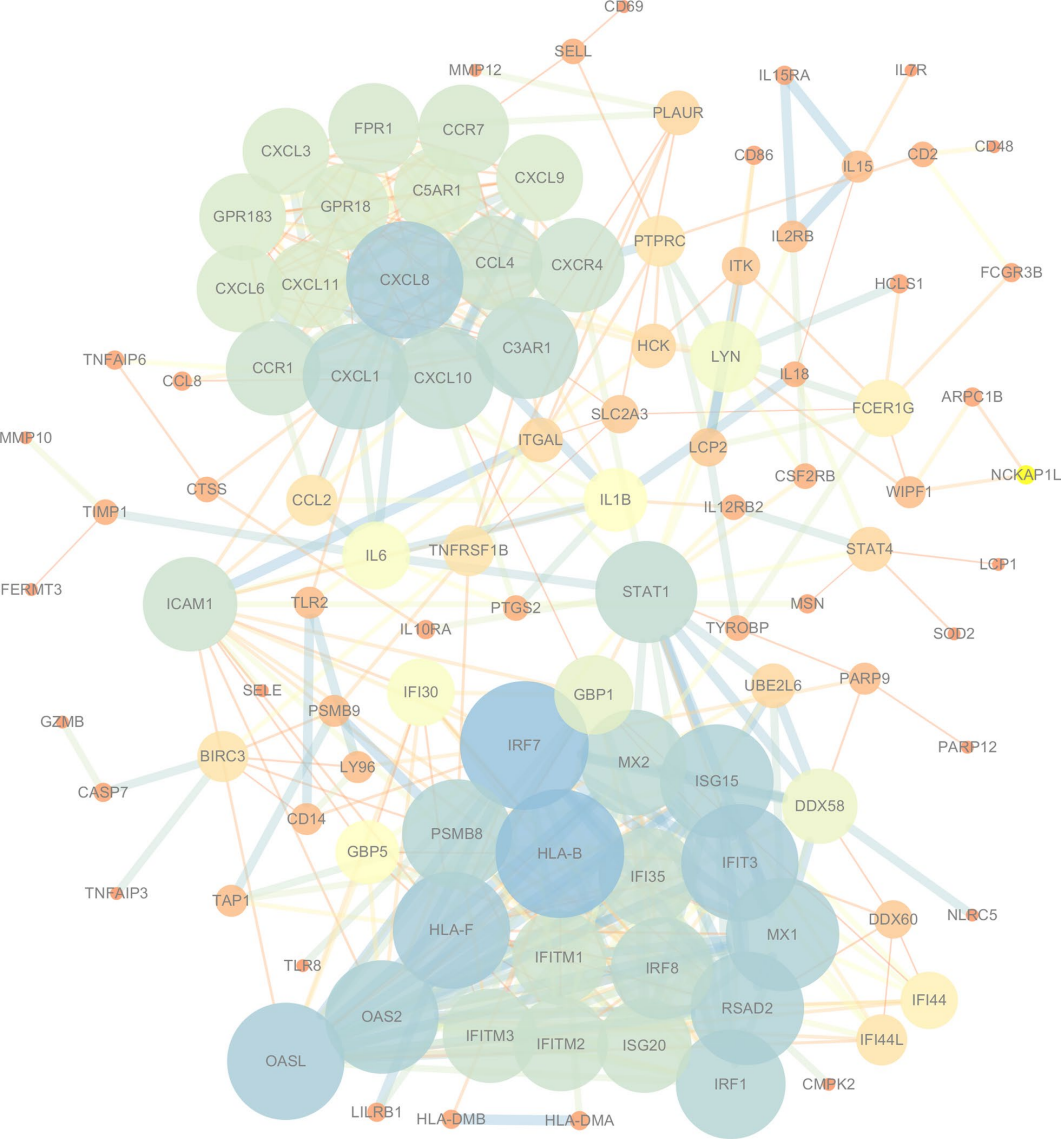
The protein–protein interaction (PPI) network of enriched DEGs

After merging the DEGs involved in the relevant pathways of the Turquoise module through the GSEA enrichment analysis, we constructed the protein–protein interaction (PPI) network of enriched DEGs based on the String database (Fig. 10). Table 3 lists the network parameters of top 20 DEGs, such as HLA-B, IRF7, HLA-F, IFIT3, OASL, CXCL8, OAS2, MX1, ISG15, RSAD2, IRF1, etc.

**Table 3:** The parameters of node in the PPI network

Node	Degree	DMNC	EPC	Closeness	Betweenness	Stress	Clustering coefficient
HLA-B	26	0.80384	51.600	54.68333	960.1928	4462	0.51385
IRF7	26	0.77479	51.902	56.48333	909.1212	5070	0.49538
HLA-F	23	0.80384	51.638	52.41667	222.4021	2268	0.65613
IFIT3	23	0.81353	51.946	53.65000	316.7897	2992	0.66403
OASL	23	0.76995	51.767	52.58333	155.9646	1834	0.62846
CXCL8	23	0.59562	49.514	54.95000	1155.311	4654	0.48617
OAS2	22	0.79905	51.740	51.91667	124.6154	1626	0.66234
MX1	22	0.83561	51.744	53.15000	229.5482	2374	0.69264
ISG15	22	0.83561	51.509	53.15000	229.5482	2374	0.69264
RSAD2	22	0.86481	51.910	48.93333	280.9047	2708	0.66234
IRF1	21	0.85350	51.580	53.48333	231.3252	2974	0.71905
PSMB8	21	0.78567	51.550	50.73333	725.2455	3582	0.66190
MX2	20	0.92117	51.595	52.15000	166.9635	1952	0.78947
CXCL1	20	0.83742	49.030	48.90000	448.4261	2204	0.60526
IRF8	19	0.84429	51.163	49.66667	71.51968	1218	0.73684
STAT1	19	0.45368	49.415	56.78333	3186.268	16,486	0.19883
CXCL10	19	0.74378	48.905	52.70000	828.0069	5742	0.64912
C3AR1	19	0.75048	48.447	49.28333	510.6841	1548	0.65497
IFI35	18	0.99902	51.134	46.76667	16.85864	158	0.88889
IFITM2	17	1.07668	51.137	46.26667	0.740030	16	0.97794
IFITM1	17	1.07668	50.947	46.26667	0.740030	16	0.97794
IFITM3	17	1.07668	51.047	46.26667	0.740030	16	0.97794
ISG20	17	1.07668	50.755	46.26667	0.740030	16	0.97794
ICAM1	17	0.81806	49.857	53.95000	1480.959	9140	0.34559
CCL4	17	0.87430	48.431	47.06667	79.97945	364	0.79412
CXCR4	17	0.95127	48.669	50.70000	588.8160	3532	0.77941
CCR1	17	0.88240	48.938	46.43333	61.21627	226	0.80147
FPR1	16	0.95127	48.054	46.10000	82.02197	356	0.88333
CCR7	16	1.05156	48.012	46.10000	237.2305	1468	0.87500
CXCL9	15	1.05156	47.915	45.43333	0.000000	0	1.00000

EPC, Edge Percolated Component; MNC, Maximum Neighborhood Component; DMNC, Density of Maximum Neighborhood Component, EcCentricity, Closeness, Betweenness, and Stress

**Fig. 11:**
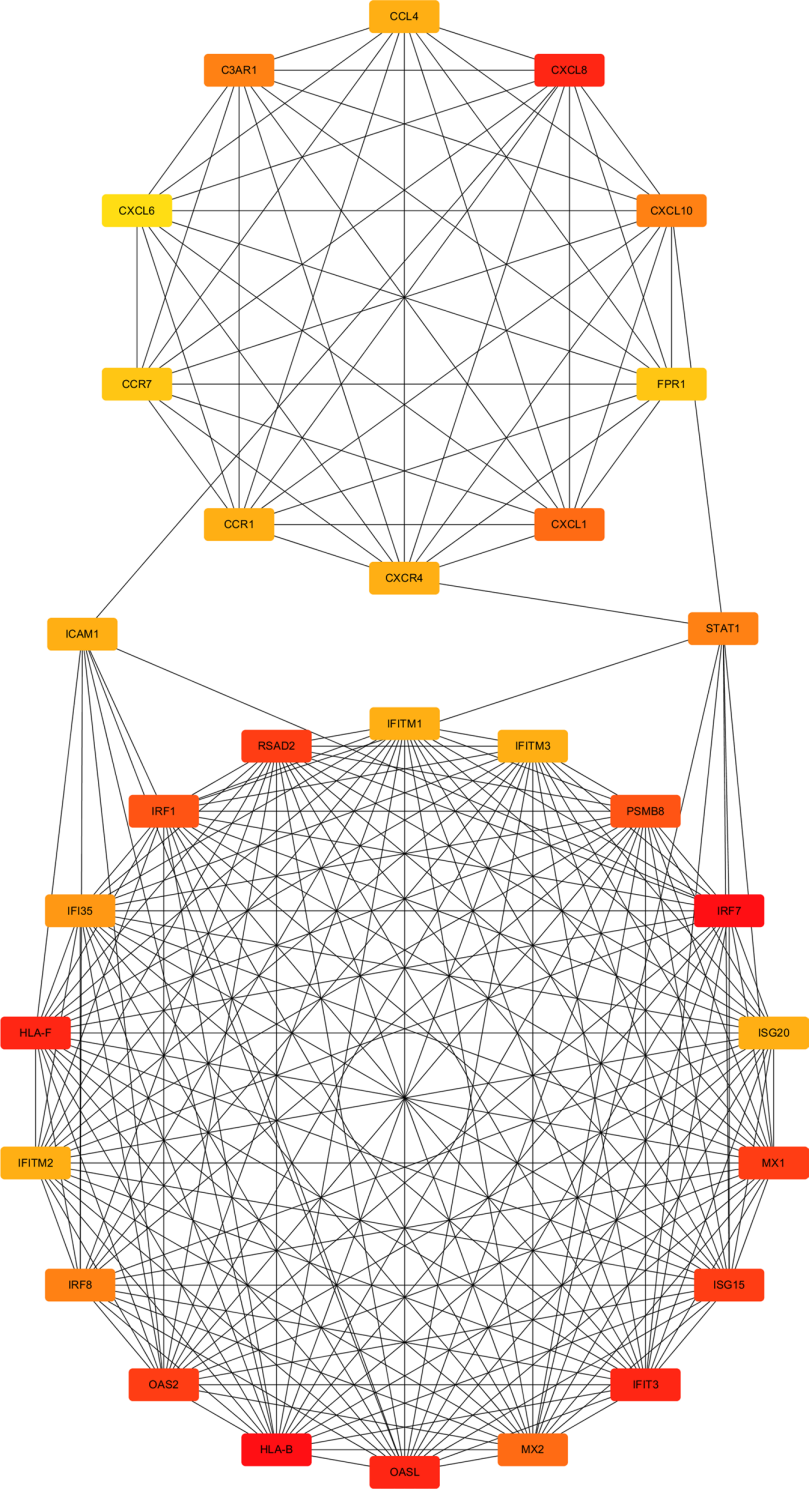
The Clusters of PPI network determined by MCODE module

As show in Fig. 11, the MCODE module determines two clusters (highly interconnected regions) in the network, with score = 17.647 and 10, respectively. Clusters in a protein–protein interaction network are often protein complexes and parts of pathways, which mean different things in different types of networks. Interestingly, cluster 1 and cluster 2 are linked by STAT1 and ICAM1. Current studies have shown that ICAM1 is a downstream gene of STAT1, and activation of STAT1 induces the expression of ICAM1 [25]. Based on the scoring parameters in Table 4, simultaneously combine the comprehensive analysis of signaling information network and miRNA-gene interaction network we constructed before, it is speculated that STAT1 may be a key gene affecting the process of RAS.

**Table 4:** The pharmacokinetic characteristics of potential natural compounds for RAS treatment

Molecule name	MW	AlogP	Hdon	Hacc	OB (%)	Caco-2	BBB	DL	FASA-	TPSA	RBN
Genistein	270.25	2.07	3	5	17.93	0.43	− 0.40	0.21	0.00	90.90	1
Daidzein	254.25	2.33	2	4	19.44	0.59	− 0.22	0.19	0.00	70.67	1
Kaempferol	286.25	1.77	4	6	41.88	0.26	− 0.55	0.24	0.00	111.1	1
Resveratrol	228.26	3.01	3	3	19.07	0.80	− 0.01	0.11	0.49	60.69	2
Rosmarinic acid	360.34	2.69	5	8	1.38	− 0.54	− 1.24	0.35	0.47	144.5	7
Triptolide	360.44	0.87	1	6	51.29	0.25	− 0.19	0.68	0.28	84.12	1
Quercetin	302.25	1.50	5	7	46.43	0.05	− 0.77	0.28	0.38	131.4	1
(-)-Epigallocatechin-3-gallate	458.40	2.89	8	11	55.09	− 0.57	− 1.70	0.77	0.37	197.4	4

MW, molecular weight; AlogP, the critical for measuring hydrophobicity of molecule; Hdon and Hacc, the measures of the hydrogen-bonding ability of a molecule expressed in terms of number of possible hydrogen-bond donors and acceptors, respectively; OB, Oral bioavailability; Caco-2, the ingredients’ transport rates (nm/s) in Caco-2 monolayers to represent the intestinal epithelial permeability; BBB, blood–brain barrier; DL, drug-likeness, a qualitative concept used in drug design for an estimate on how “drug-like” a prospective compound is; FASA-, fractional water accessible surface area of all atoms with negative partial charge, can be used as drug-likeness evaluation for drug-like molecules; TPSA, a physico chemical property describing the polarity of molecules; RBN, description for molecular flexibility, the number of bonds which allow free rotation around themselves, and roughly proportional to molecular size for many “drug-like” compounds

### Screening of potential natural compounds

Finally, eight natural components were finally screened to have high affinity with STAT1 based on the TCMSP database, including genistein, daidzein, kaempferol, resveratrol, rosmarinic acid, triptolide, quercetin and (-)-epigallocatechin-3-gallate. Table 4 shows the pharmacokinetic characteristics of above ingredients.

### Predict potential targets based on Pharmmapper and enrichment analysis

**Fig. 12:**
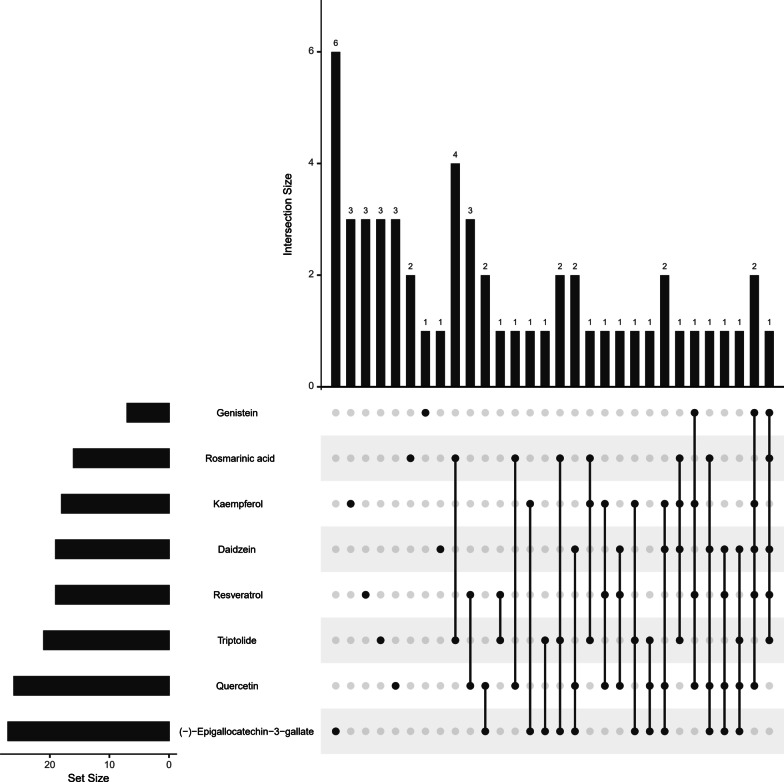
The intersection of the potential targets of 8 compounds

We predicted the potential targets of the above 8 compounds based on Pharmmapper. The Table 5 lists the intersection of predicted targets and DEGs of RAS, and Fig. 12 indicates that the potential targets of these compounds affecting RAS are very similar, and it also suggests that these compounds may affect the process of RAS through multiple targets.

**Table 5:** The predicted targets of RAS treatment for each compound

Molecule	Gene
Genistein	STAT1/ICAM1/VCAM1/SOD2/CRYAB/C3/SELE
Daidzein	STAT1/ICAM1/VCAM1/SOD2/IL6/C3/CAMK4/CSF2RB/HCK/HLA-B/HLA-E/IFNGR1/INHBA/LTF/MSN/NT5C3A/PANK1/THBS1/TPK1
Quercetin	STAT1/ICAM1/VCAM1/SELE/CYP1B1/NQO1/IRF1/CAMK4/CSF2RB/HCK/HLA-B/HLA-E/HLA-G/HMGCS1/IFNGR1/LTF/LYZ/MMP1/MMP9/NPR3/NT5C3A/PANK1/THBS1/TLR1/MMP3/IL6
Kaempferol	STAT1/ICAM1/VCAM1/SELE/CYP1B1/C3/CD74/GZMB/HLA-E/IFNGR1/IL10RA/LTF/MS4A1/MSN/THBS1/THBS2/TPK1/WARS1
Resveratrol	IL6/STAT1/ICAM1/SELE/VCAM1/SOD2/CYP1B1/NQO1/BIRC3/C1R/C3/CSF2RB/HCK/HLA-E/ME1/MSN/NPR3/SAMHD1/TLR1
(-)-Epigallocatechin-3-gallate	IL6/MMP3/STAT1/TLR4/BTK/C3/CAMK4/FAP/GCH1/GLUL/GZMB/GZMK/HCK/HLA-B/HLA-E/HMGCS1/IFNGR1/KLF10/LTF/MMP1/MSN/NT5C3A/PANK1/PLA2G2A/THBS1/TRIM21/WARS1
Rosmarinic Acid	STAT1/IDO1/ALOX12/ANXA1/BTK/CD38/HCK/HLA-E/LAP3/LYZ/MS4A1/NT5C3A/RGS18/SOD2/TPK1/TRIM21
Triptolide	STAT1/CXCR4/BIRC3/CCR7/ALOX12/ANXA1/BTK/CAMK4/GBP1/HLA-E/HMGCS1/LAP3/LTF/MS4A1/MSN/PLA2G2A/RGS18/SOD2/TPK1/TRIM21/WARS1

**Fig. 13:**
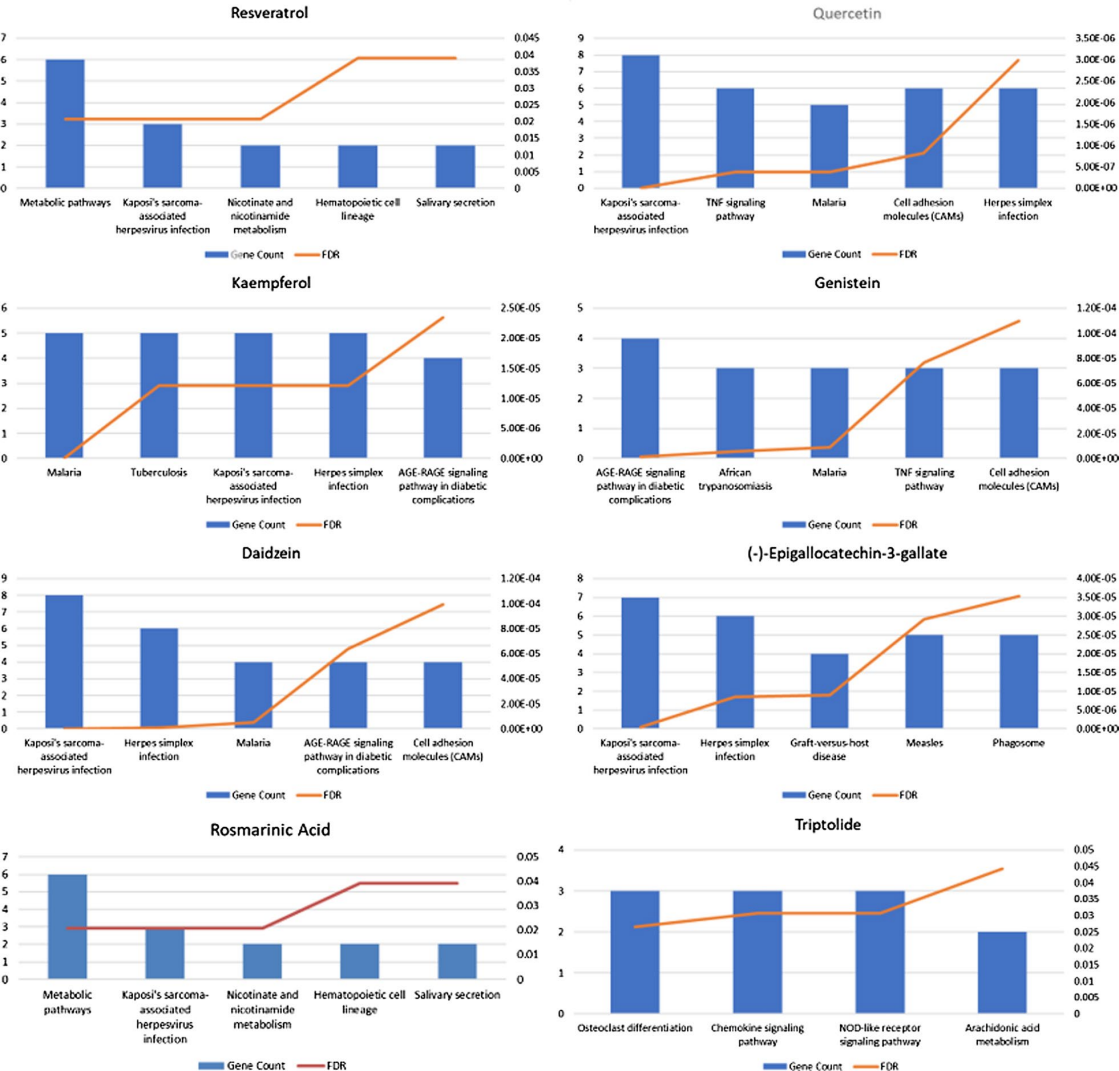
The result of KEGG enrichment

Figure 13 shows the results of the KEGG enrichment analysis on the predicted targets of these compounds, suggesting that the potential targets of these compounds to affect the RAS process are mainly concentrated in a variety of viral infection-related immune pathways, TNF pathways, cell adhesion and other biological pathways.

### Confirm the target of RAS based on reverse docking technology

**Fig. 14:**
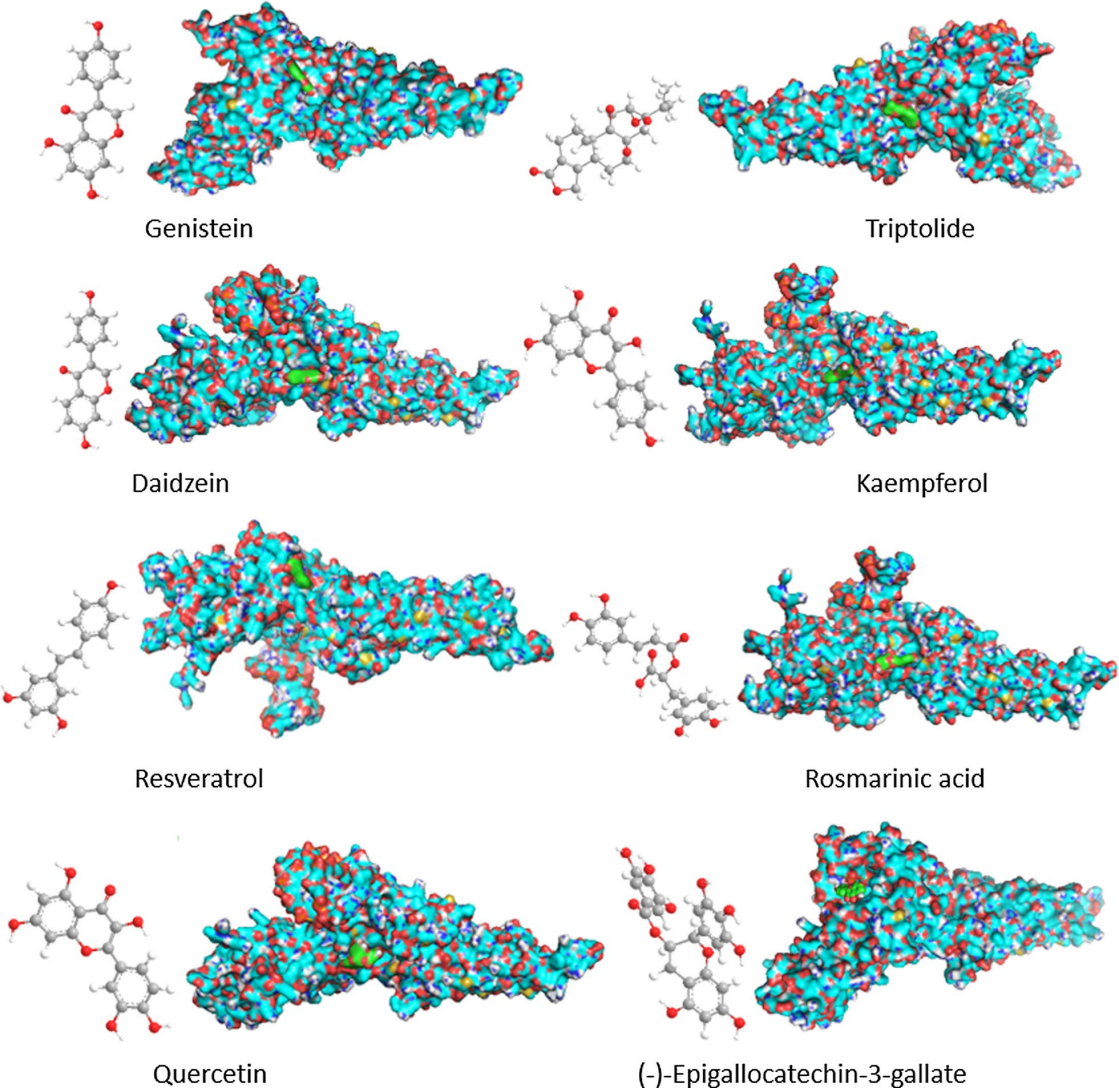
Interaction between STAT1 and inhibitors (genistein, daidzein, kaempferol, resveratrol, rosmarinic acid, triptolide, quercetin, and (-)-epigallocatechin-3-gallate)

As shown in Fig. 14, the results of reverse molecular docking suggest that different compounds may bind to different parts of the STAT1 protein, while compounds with similar molecular structures bind to similar positions of the STAT1 protein. The higher the docking score, the stronger the binding force to the protein. According to the docking score, it is sorted from high to low: triptolide (− 9.1 kcal/mol), (-)-epigallocatechin-3-gallate (− 8.1 kcal/mol), rosmarinic acid (− 7.3 kcal/mol), quercetin (− 7.3 kcal/mol), genistein (− 7.1 kcal/mol), daidzein (− 6.9 kcal/mol), kaempferol (− 6.9 kcal/mol), resveratrol (− 5.6 kcal/mol).

## Discuss

In this article, we initially screened 12,548 differentially expressed genes in ulcer tissues and normal tissues of RAS patients through differentially analysis, and finally included 187 genes in the study according to the screening criteria. After we constructed the miRNA-gene interaction network and signaling information network of these genes, we determined the candidate key node targets. Then we applied WGCNA to cluster analysis of all genes and found that the key node targets we were interested in were all in the same module, so we carried out a further enrichment analysis for the differential genes in this module, and further screened out the hub genes. Then, functional analysis of hub genes in modules of interest was performed, which indicated that such hub genes were mainly involved in pathways related to immune response, virus infection, epithelial cell, signal transduction. The PPI network was identified, and two modules linked through STAT1 were identified. It was finally determined that multiple biological pathways mediated by STAT1 may affect the process of RAS disease. It is speculated that STAT1 may become a potential target of RAS treatment. Finally, the molecular reverse docking technology was used to screen out several compounds that may act on the STAT1 protein. Several of these compounds have been confirmed as inhibitors of the STAT1 protein, and they are expected to become potential therapeutic drugs for RAS.

Many factors have already been implicated in the promotion and/or exacerbation of RAS. However, the principal etiology of RAS still remains unclear. Considerable research attention has been devoted to elucidating the causes of RAS, several factors have been proposed as possible causative agents. The potential etiopathogenic agents include local and systemic conditions, positive family history, trauma in individuals who are genetically susceptible to RAS (certain genetically specific HLAs have been identified in RAS patients), nutritional factors (such as deficiency of folate and B-complex vitamins), immunologic factors, psychosocial stress, and allergy to dietary constituents, local trauma, nutritional deficiency, food hypersensitivity, smoking cessation, and psychological stress, and infectious microbial factors, etc. [2, 3, 6].

For the past 20 years, extensive research has focused predominantly on immunologic factors, but it is evident that there is no unifying theory of the immunopathogenesis of RAS [3]. Larger parts of the study on the cause of RAS, which demonstrated a connection shared by a small number of immune-mediated respons as well as the development of RAS, are made up of the cytotoxic action of T lymphocytes as well as monocytes on the oral epithelium, immune complex vasculitis, antibody dependent cell-mediated cytotoxicity, in addition to the drawbacks in lymphocyte subpopulations. Various immune reactions have led todamages which were brought about from the deposition of immune complexes in the oral epithelium. However, the trigger for these responses is still less explicit [2, 4, 7]. Researches have revealed a RAS severity’s relevance to abnormal scales of CD4+ and CD8+ cells, changes of the CD4+ :CD8+ rate, in virtue of elevating the levels of interleukin 2, interferon gamma, coupled with tumor necrosing factor-α (TNF-α) mRNA in RAS lesions [3–6]. Peripheral blood mononuclear cells of RAS patients have been revealed oriented with secrete great deal of TNF-α, which symbolizes the indispensable role of TNF in the aspect of RAS pathogenesis. In consequence, TNF-α-mediated endothelial cell adhesion and neutrophil chemotaxis are working as an initiator of the cascade of inflammatory procedures which is resulting in ulceration [5]. A large majority of the TNF-α is made to respond to excitation of toll-like receptors (TLRs), which is a series of functional membrane receptors in relevance to and safeguarding for epithelial barrier featured by not only pro- but also anti-inflammatory properties [4]. Since levels of serum immunoglobulins and natural killer cells exert essential role in normal limited range in RAS patients, attention has been paid to a dysregulated, local, cellmediated immune response of benefit to accumulation of subsets of T cells [6]. The local immune response leads to final tissue resession manifesting as RAS.

We used the MCODE module in crytoscape 3.7.3 to identify two core Cluster modules in the enriched differential expression genes. As shown in Fig. 11, Cluster 1 and Cluster 2 was linked by STAT1 and ICAM1, while ICAM1 was a downstream protein of STAT1, the phosphorylation degree of STAT1 could affect the expression of ICAM1 protein [26]. It was not difficult to find that the initial factors that affect the pathogenesis of RAS is mainly concentrated in interferon pathways, including interferon regulatory factor (IRF), interferon gene promoters and interferon stimulation response genes (ISG), or involve viruses Infection causes an anti-viral protein such as IFITMs, OAS2 or OASL, which was also activated by the interferon pathway. In addition, Cluster1 also included RAS patient-specific expression gene such as HLA-B or HLA-F. While Cluster 2 mainly contained chemokines and their receptors. We believe that the interferon route activated the chemokine and its receptor through the STAT1 protein, the crosstalk between the matrix metalloprotease system and the chemokine network had been proved, and chemokines and their receptors may regulate the activity of matrix metalloproteinases [28, 29], which may affect the synthesis and degradation of oral epithelial collagen, and finally exhibited in the form of ulcers. According to previous studies and the DEGs in this chip, many types of matrix metalloproteins (MMPs) or tissue inhibitor of metalloproteinases (TIMPs) in RAS have been confirmed to be differentially expressed compared with normal tissues [30–32]. In addition, ICAM1 can also mediate synthesis and decomposition of collagen, which also requires STAT1 mediation. Therefore, inhibition of STAT1 may cut off some abnormalities in the interferon pathway and inhibit chemokines activity, which in turn affects the related activities of matrix metalloproteinases and affects the synthesis or decomposition of collagen in the oral cavity, and may also be one of the mechanisms of RAS.

In addition, the latest research had confirmed that the levels of Galectin and IL-6 in the serum or saliva of patients with periodontitis have changed significantly [33, 34], suggesting that these factors may be closely related to oral diseases. Interestingly, the Galectin pathway may also mediate the progression of RAS disease, which may be another biological pathway completely different from STAT1 mediated pathway. In this study, the expression of Galectin-1, Galectin-2 and Galectin-3 in the ulcer tissues of RAS patients also changed significantly. Among them, Galectin-1 was closely related to excessive inflammation, mainly through regulating T cells, B cells, macrophages and granulocytes and other immune cells to promote immune tolerance and down-regulate innate and adaptive immune responses [35]; Galectin-3 not only affected the synthesis of type I collagen, but also affected the activity of TIMPs and MMPs [36]. The most noteworthy thing was that the expression of IL-6 in the ulcer tissue of RAS patients had changed significantly. IL-6 was the key node gene in the signaling information network and miRNA-gene interaction network we constructed before. The enrichment analysis also showed that IL-6 participated in multiple biological pathways. At the same time, IL-6 was also one of the key factors to activate STAT1 [37], and the role of IL-6-related biological pathways mediated by STAT1 in the progression of RAS was also worthy of further in-depth study.

Finally, we have also confirmed that STAT1 protein is one of the potential therapeutic targets of RAS, and this target can be used to screen potential therapeutic compounds. Finally, genistein, daidzein, kaempferol, resveratrol, rosmarinic acid, triptolide, quercetin and (-)-epigallocatechin-3-gallate were selceted from the TCMSP databse, and both of them is the STAT-1 inhibitor [38–42]. Interestingly, some of those ingredients, such as rosmarinic acid, quercetin, (-)-Epigallocatechin-3-gallate, resveratrol, etc., have already been made into topical formulations for the treatment of oral ulcers, such as quercetin, (-)-Epigallocatechin-3-gallate, resveratrol [43–45]. The results of reverse molecular docking suggest that in addition to triptolide, (-)-Epigallocatechin-3-gallate and resveratrol, the other 5 compounds (flavonoids) with similar structures bind to STAT1 at almost the same position, that is, this position may be It is the key position for flavonoids to inhibit stat1 protein.

## Conclusions

We identified potential biomarkers that might contribute to the diagnosis and treatment of RAS based on WGCNA, it was speculated that STAT1 is one of the potential therapeutic targets. The results of reverse molecular docking suggested that we can screen RAS therapeutic drugs from STAT-1 inhibitors.

## Data Availability

The datasets used or analysed during the current study are available from the corresponding author on reasonable request.

## Abbreviations

RAS: The recurrent aphthous stomatitis
NCBI: National Center for Biotechnology Information
GEO: Gene Expression Omnibus
WGCNA: Weighted Gene Co-Expression Network Analysis
TOM: Topological Overlap Matrix
STAT1: Signal transducer and activator of transcription 1
ICAM1: Intercellular cell adhesion molecule-1
TCMSP: Traditional Chinese Medicine Systems Pharmacology Database and Analysis Platform
DEGs: Differential expression genes
GSEA: Gene Set Enrichment Analysis
GO: Gene ontology
KEGG: Kyoto encyclopedia of genes genomes
PPI: Protein–protein interactions
MCODE: Molecular Complex Detection
PCA: Principal component analysis
MW: Molecular weight
AlogP: The critical for measuring hydrophobicity of molecule
OB: Oral bioavailability
BBB: Blood–brain barrier
DL: Drug-likeness
FASA-: Fractional water accessible surface area of all atoms with negative partial charge
TPSA: A physico chemical property describing the polarity of molecules
RBN: Description for molecular flexibility, the number of bonds which allow free rotation around themselves
EPC: Edge Percolated Component
MNC: Maximum Neighborhood Component
DMNC: Density of Maximum Neighborhood Component
TLRs: Toll-like receptors
TNF: Tumor necrosing factor
IRF: Interferon regulatory factor
ISG: Interferon stimulation response genes
MMPs: Matrix metalloproteins
TIMPs: Tissue inhibitor of metalloproteinases
IL-6: Interleukin-6
HLA: Human leukocyte antigen
CD4: cluster of differentiation 4
CD8: cluster of differentiation 8
